# *trans*-Dichloridobis(2,2-dimethyl­prop­ane-1,3-diamine-κ^2^ *N*,*N*′)chromium(III) perchlorate

**DOI:** 10.1107/S1600536808032911

**Published:** 2008-10-18

**Authors:** Jong-Ha Choi, Sang Hak Lee, Uk Lee

**Affiliations:** a Department of Chemistry, Andong National University, Andong 760-749, Republic of Korea; b Department of Chemistry, Kyungpook National University, Daegu 702-701, Republic of Korea; c Department of Chemistry, Pukyong National University, 599-1 Daeyeon 3-dong Nam-gu, Busan 608-737, Republic of Korea

## Abstract

In the title salt, [CrCl_2_(C_5_H_14_N_2_)_2_]ClO_4_, the Cr atom is in a *trans*-CrCl_2_N_4_ octa­hedral environment comprising the four N atoms of two chelating 2,2-dimethyl­propane-1,3-diamine ligands and two Cl atoms. The two six-membered CrC_3_N_2_ rings in the cation adopt *anti* chair–chair conformations with respect to each other. The perchlorate anion is disordered over two positions in respect of the Cl and an O atom in a 6:4 ratio. N—H⋯O hydrogen bonds link the cations and anions into a layer structure.

## Related literature

For the synthesis, see: House (1986 ▶). For related structures, see: Choi *et al.* (2002 ▶, 2007 ▶). For the spectroscopic properties, see: Choi (2000 ▶); Poon & Pun (1980 ▶).
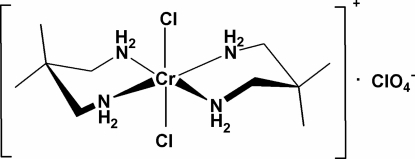


## Experimental

### 

#### Crystal data

[CrCl_2_(C_5_H_14_N_2_)_2_]ClO_4_
*M* *_r_* = 426.71
Monoclinic, 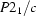

*a* = 6.6373 (6) Å
*b* = 20.767 (2) Å
*c* = 13.878 (2) Å
β = 100.249 (9)°
*V* = 1882.4 (4) Å^3^
*Z* = 4
Mo *K*α radiation
μ = 1.05 mm^−1^
*T* = 298 (2) K
0.32 × 0.30 × 0.25 mm

#### Data collection

Stoe Stadi-4 diffractometer
Absorption correction: numerical (*X-SHAPE*; Stoe & Cie, 1996 ▶) *T*_min_ = 0.805, *T*_max_ = 0.942
4305 measured reflections
4305 independent reflections
3453 reflections with *I* > 2σ(*I*)
3 standard reflections frequency: 60 min intensity decay: 2.7%

#### Refinement

*R*[*F* ^2^ > 2σ(*F* ^2^)] = 0.051
*wR*(*F* ^2^) = 0.146
*S* = 1.11
4305 reflections
217 parameters
H-atom parameters constrained
Δρ_max_ = 0.54 e Å^−3^
Δρ_min_ = −0.81 e Å^−3^

### 

Data collection: *STADI-4* (Stoe & Cie, 1996 ▶); cell refinement: *STADI-4*; data reduction: *X-RED* (Stoe & Cie, 1996 ▶); program(s) used to solve structure: *SHELXS97* (Sheldrick, 2008 ▶); program(s) used to refine structure: *SHELXL97* (Sheldrick, 2008 ▶); molecular graphics: *ORTEP-3* (Farrugia, 1997 ▶); software used to prepare material for publication: *SHELXL97*.

## Supplementary Material

Crystal structure: contains datablocks global, I. DOI: 10.1107/S1600536808032911/ng2499sup1.cif

Structure factors: contains datablocks I. DOI: 10.1107/S1600536808032911/ng2499Isup2.hkl

Additional supplementary materials:  crystallographic information; 3D view; checkCIF report

## Figures and Tables

**Table 1:** Hydrogen-bond geometry (Å, °)

*D*—H⋯*A*	*D*—H	H⋯*A*	*D*⋯*A*	*D*—H⋯*A*
N1—H1*B*N⋯O2^i^	0.90	2.29	3.030 (5)	139
N2—H2*A*N⋯O3^ii^	0.90	2.23	3.099 (6)	162
N2—H2*A*N⋯O4*A*^ii^	0.90	2.42	3.183 (6)	143
N2—H2*B*N⋯O4*B*	0.90	2.36	3.217 (9)	159
N3—H3*A*N⋯O4*A*^ii^	0.90	2.60	3.482 (7)	168
N4—H4*B*N⋯O2^i^	0.90	2.14	3.030 (5)	172
N4—H4*A*N⋯O4*A*^iii^	0.90	2.54	3.403 (8)	161

Symmetry codes: (i) 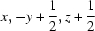; (ii) 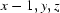; (iii) 
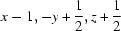
; (iv) 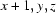.

**Fig. 1.**
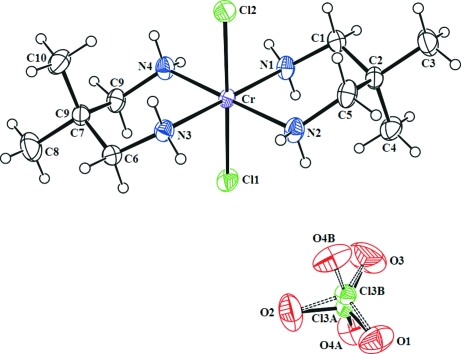
Perspective view (30% probability level) of title compound.

## supplementary crystallographic information

### Comment

The [Cr(Me_2_tn)_2_L_2_]^+^ (Me_2_tn=2,2-dimethylpropane-1,3-diamine, L = monodentate) cation can exist as *trans* and *cis* geometric isomers. Especially, there are two possible conformations with respect to the six-membered rings in the *trans* isomer. The carbon atoms of the two chelate rings of the two conformers may be on the same side (*syn* conformer) or on opposite side (*anti* conformer) of the coordination plane (Choi *et al.*,  2002; Choi *et al.*,  2007). The *syn* or *anti* conformational stereochemistry of the six-membered chelate rings can not be readily determined by spectroscopic and physicochemical methods (Poon & Pun, 1980; Choi, 2000). In order to examine the influences of counter anions and packing forces of the crystal on the conformations, we have undertaken the X-ray structural analysis of *trans*-[Cr(Me_2_tn)_2_Cl_2_]ClO_4_, (I).

The title complex has approximate *C_i_* symmetry. The two chelate rings in the complex cation are only in anti chair-chair conformation with respect to each other (Fig.1). The Cr—N and Cr—Cl bond length are very simillar to those of the *trans*-[Cr(Me_2_tn)_2_Cl_2_]Cl (Choi *et al.*, 2007). However, the significant difference between these two crystal structures is the orientations with respect to the six-membered chelate rings of two Me_2_tn ligands in the same *trans* geometry. The complex is stabilized by the formation of the extensive hydrogen bonds (Table 1).

### Experimental

The complex *trans*-[Cr(Me_2_tn)_2_Cl_2_]ClO_4_ was prepared according to the literature (House, 1986). The crystalline product deposited with ice-bath cooling was filtered off, and washed with cold 2-propanol and then diethyl ether. Recrystallization of the crude precipitate from 0.5M HCl and 70% HClO_4_ solution afforded dark green crystals suitable for X-ray analysis. Anal. Found: C, 28.02; H, 6.50; N, 13.08%. Calc. for C_10_H_28_Cl_3_CrN_4_O_4_: C, 28.15; H, 6.61; N, 13.13%.

### Refinement

H atoms were positioned geometrically and refined as riding atoms, with C—H = 0.97 (methylene), 0.96 (methyl) Å, and N—H = 0.90 Å respectively,  Uiso(H) = 1.2Ueq(C & N). The Cl and one O atoms in the perchlorate anion are disordered over two positions with site-occupancy factors fixed at 0.60 (for atoms labelled A) and 0.40 (for atoms labelled B) in the final refinement.

### Crystal data

**Table:**

[CrCl_2_(C_5_H_14_N_2_)_2_]ClO_4_	*F*(000) = 892
*M**_r_* = 426.71	*D*_x_ = 1.506 Mg m^−^^3^
Monoclinic, *P*2_1_/*c*	Mo *K*α radiation, λ = 0.71069 Å
Hall symbol: -P_2ybc	Cell parameters from 54 reflections
*a* = 6.6373 (6) Å	θ = 9.5–10.4°
*b* = 20.767 (2) Å	µ = 1.05 mm^−^^1^
*c* = 13.878 (2) Å	*T* = 298 K
β = 100.249 (9)°	Block, green
*V* = 1882.4 (4) Å^3^	0.32 × 0.30 × 0.25 mm
*Z* = 4	

### Data collection

**Table:**

Stoe Stadi-4 diffractometer	3453 reflections with *I* > 2σ(*I*)
Radiation source: fine-focus sealed tube	*R*_int_ = 0.0000
graphite	θ_max_ = 27.5°, θ_min_ = 1.8°
ω/2–θ scans	*h* = −8→8
Absorption correction: numerical (X-SHAPE; Stoe & Cie, 1996)	*k* = 0→26
*T*_min_ = 0.805, *T*_max_ = 0.942	*l* = 0→18
4305 measured reflections	3 standard reflections every 60 min
4305 independent reflections	intensity decay: 2.7%

### Refinement

**Table:**

Refinement on *F*^2^	Primary atom site location: structure-invariant direct methods
Least-squares matrix: full	Secondary atom site location: difference Fourier map
*R*[*F*^2^ > 2σ(*F*^2^)] = 0.051	Hydrogen site location: inferred from neighbouring sites
*wR*(*F*^2^) = 0.146	H-atom parameters constrained
*S* = 1.11	*w* = 1/[σ^2^(*F*_o_^2^) + (0.0733*P*)^2^ + 1.4729*P*] where *P* = (*F*_o_^2^ + 2*F*_c_^2^)/3
4305 reflections	(Δ/σ)_max_ < 0.001
217 parameters	Δρ_max_ = 0.54 e Å^−^^3^
0 restraints	Δρ_min_ = −0.81 e Å^−^^3^

### Special details

**Table:**

Geometry. All esds (except the esd in the dihedral angle between two l.s. planes) are estimated using the full covariance matrix. The cell esds are taken into account individually in the estimation of esds in distances, angles and torsion angles; correlations between esds in cell parameters are only used when they are defined by crystal symmetry. An approximate (isotropic) treatment of cell esds is used for estimating esds involving l.s. planes.
Refinement. Refinement of F^2^ against ALL reflections. The weighted R-factor wR and goodness of fit S are based on F^2^, conventional R-factors R are based on F, with F set to zero for negative F^2^. The threshold expression of F^2^ > 2sigma(F^2^) is used only for calculating R-factors(gt) etc. and is not relevant to the choice of reflections for refinement. R-factors based on F^2^ are statistically about twice as large as those based on F, and R- factors based on ALL data will be even larger.

### Fractional atomic coordinates and isotropic or equivalent isotropic displacement parameters (Å^2^)

**Table:**

	*x*	*y*	*z*	*U*_iso_*/*U*_eq_	Occ. (<1)
Cr	0.43254 (6)	0.23278 (2)	0.57694 (3)	0.03056 (15)	
Cl1	0.67222 (13)	0.25634 (5)	0.47898 (7)	0.0540 (2)	
Cl2	0.18895 (12)	0.21076 (4)	0.67312 (7)	0.0493 (2)	
Cl3A	0.9541 (3)	0.11988 (10)	0.31249 (14)	0.0471 (4)	0.60
Cl3B	0.8697 (4)	0.11095 (15)	0.3227 (2)	0.0434 (6)	0.40
O4B	0.6845 (14)	0.1083 (5)	0.3541 (7)	0.096 (3)	0.40
O4A	1.1593 (9)	0.1344 (3)	0.3048 (5)	0.0913 (18)	0.60
O1	0.8766 (6)	0.06609 (19)	0.2505 (3)	0.0957 (12)	
O2	0.8510 (7)	0.1748 (2)	0.2793 (3)	0.1124 (15)	
O3	0.9840 (9)	0.1052 (3)	0.4093 (3)	0.140 (2)	
N1	0.6658 (4)	0.20091 (13)	0.6894 (2)	0.0420 (6)	
H1AN	0.7834	0.2010	0.6656	0.050*	
H1BN	0.6795	0.2305	0.7375	0.050*	
N2	0.4181 (4)	0.13895 (13)	0.5210 (2)	0.0466 (7)	
H2AN	0.2998	0.1354	0.4782	0.056*	
H2BN	0.5200	0.1348	0.4865	0.056*	
N3	0.1983 (4)	0.26418 (12)	0.46576 (19)	0.0385 (6)	
H3AN	0.1880	0.2354	0.4167	0.046*	
H3BN	0.0806	0.2622	0.4893	0.046*	
N4	0.4506 (4)	0.32609 (13)	0.6350 (2)	0.0448 (6)	
H4AN	0.3526	0.3297	0.6719	0.054*	
H4BN	0.5717	0.3295	0.6758	0.054*	
C1	0.6476 (5)	0.13679 (16)	0.7353 (2)	0.0444 (7)	
H1A	0.5282	0.1372	0.7667	0.053*	
H1B	0.7668	0.1302	0.7860	0.053*	
C2	0.6295 (5)	0.07995 (15)	0.6641 (3)	0.0411 (7)	
C3	0.6162 (7)	0.01867 (19)	0.7255 (3)	0.0644 (11)	
H3A	0.6049	−0.0184	0.6836	0.077*	
H3B	0.4982	0.0212	0.7565	0.077*	
H3C	0.7374	0.0151	0.7746	0.077*	
C4	0.8169 (6)	0.07509 (19)	0.6154 (3)	0.0569 (9)	
H4A	0.8011	0.0393	0.5708	0.068*	
H4B	0.9368	0.0688	0.6645	0.068*	
H4C	0.8311	0.1141	0.5801	0.068*	
C5	0.4308 (5)	0.08283 (16)	0.5890 (3)	0.0495 (8)	
H5A	0.4181	0.0435	0.5507	0.059*	
H5B	0.3161	0.0846	0.6235	0.059*	
C6	0.2096 (5)	0.32895 (16)	0.4223 (2)	0.0441 (7)	
H6A	0.0874	0.3356	0.3735	0.053*	
H6B	0.3261	0.3300	0.3889	0.053*	
C7	0.2293 (5)	0.38464 (15)	0.4953 (2)	0.0410 (7)	
C8	0.2337 (8)	0.4469 (2)	0.4358 (4)	0.0715 (12)	
H8A	0.2455	0.4833	0.4791	0.086*	
H8B	0.1095	0.4503	0.3886	0.086*	
H8C	0.3488	0.4459	0.4026	0.086*	
C9	0.4311 (5)	0.38267 (15)	0.5681 (3)	0.0469 (8)	
H9A	0.5435	0.3821	0.5320	0.056*	
H9B	0.4433	0.4217	0.6071	0.056*	
C10	0.0472 (6)	0.38715 (19)	0.5480 (3)	0.0571 (9)	
H10A	0.0640	0.4224	0.5935	0.069*	
H10B	0.0388	0.3475	0.5827	0.069*	
H10C	−0.0762	0.3932	0.5010	0.069*	

### Atomic displacement parameters (Å^2^)

**Table:**

	*U*^11^	*U*^22^	*U*^33^	*U*^12^	*U*^13^	*U*^23^
Cr	0.0243 (2)	0.0293 (2)	0.0375 (3)	0.00052 (16)	0.00378 (17)	−0.00218 (18)
Cl1	0.0375 (4)	0.0574 (5)	0.0726 (6)	0.0075 (4)	0.0244 (4)	0.0073 (4)
Cl2	0.0377 (4)	0.0561 (5)	0.0578 (5)	0.0062 (3)	0.0185 (4)	0.0123 (4)
Cl3A	0.0561 (12)	0.0449 (9)	0.0375 (8)	0.0080 (9)	0.0011 (9)	−0.0001 (6)
Cl3B	0.0381 (13)	0.0507 (14)	0.0405 (12)	0.0016 (11)	0.0046 (11)	0.0027 (9)
O4B	0.077 (5)	0.122 (8)	0.096 (6)	−0.025 (5)	0.034 (5)	−0.052 (6)
O4A	0.065 (3)	0.100 (4)	0.099 (4)	−0.011 (3)	−0.013 (3)	0.000 (4)
O1	0.110 (3)	0.091 (3)	0.092 (2)	−0.029 (2)	0.034 (2)	−0.038 (2)
O2	0.126 (3)	0.077 (2)	0.122 (3)	0.025 (2)	−0.010 (3)	0.024 (2)
O3	0.167 (5)	0.178 (5)	0.062 (2)	0.004 (4)	−0.019 (3)	0.017 (3)
N1	0.0319 (12)	0.0373 (13)	0.0526 (16)	0.0014 (10)	−0.0036 (11)	−0.0012 (12)
N2	0.0489 (15)	0.0369 (14)	0.0502 (16)	0.0038 (12)	−0.0017 (12)	−0.0068 (12)
N3	0.0332 (12)	0.0391 (14)	0.0420 (14)	0.0029 (10)	0.0031 (10)	−0.0021 (11)
N4	0.0485 (15)	0.0358 (13)	0.0460 (15)	0.0014 (11)	−0.0029 (12)	−0.0048 (12)
C1	0.0439 (17)	0.0408 (17)	0.0467 (17)	0.0064 (13)	0.0035 (14)	0.0064 (14)
C2	0.0373 (15)	0.0323 (15)	0.0554 (19)	0.0035 (12)	0.0126 (13)	0.0059 (13)
C3	0.070 (3)	0.044 (2)	0.084 (3)	0.0058 (18)	0.024 (2)	0.0195 (19)
C4	0.0474 (19)	0.053 (2)	0.076 (3)	0.0068 (16)	0.0259 (18)	0.0017 (19)
C5	0.0434 (17)	0.0322 (16)	0.071 (2)	−0.0017 (13)	0.0040 (16)	−0.0052 (15)
C6	0.0467 (17)	0.0487 (18)	0.0371 (16)	0.0131 (14)	0.0080 (13)	0.0041 (14)
C7	0.0417 (16)	0.0352 (15)	0.0479 (17)	0.0068 (12)	0.0133 (13)	0.0069 (13)
C8	0.083 (3)	0.045 (2)	0.086 (3)	0.007 (2)	0.015 (2)	0.022 (2)
C9	0.0448 (18)	0.0321 (15)	0.064 (2)	−0.0009 (13)	0.0094 (16)	−0.0003 (14)
C10	0.049 (2)	0.052 (2)	0.077 (3)	0.0090 (16)	0.0259 (19)	−0.0037 (18)

### Geometric parameters (Å, °)

**Table:**

Cr—N3	2.090 (3)	C1—H1A	0.9700
Cr—N2	2.093 (3)	C1—H1B	0.9700
Cr—N4	2.094 (3)	C2—C4	1.522 (4)
Cr—N1	2.100 (3)	C2—C5	1.530 (5)
Cr—Cl2	2.3179 (9)	C2—C3	1.543 (5)
Cr—Cl1	2.3212 (9)	C3—H3A	0.9600
Cl3A—Cl3B	0.629 (2)	C3—H3B	0.9600
Cl3A—O3	1.357 (4)	C3—H3C	0.9600
Cl3A—O2	1.368 (4)	C4—H4A	0.9600
Cl3A—O4A	1.418 (6)	C4—H4B	0.9600
Cl3A—O1	1.447 (4)	C4—H4C	0.9600
Cl3B—O3	1.308 (5)	C5—H5A	0.9700
Cl3B—O1	1.374 (5)	C5—H5B	0.9700
Cl3B—O4B	1.377 (9)	C6—C7	1.528 (5)
Cl3B—O2	1.453 (5)	C6—H6A	0.9700
N1—C1	1.490 (4)	C6—H6B	0.9700
N1—H1AN	0.9000	C7—C10	1.520 (5)
N1—H1BN	0.9000	C7—C9	1.528 (5)
N2—C5	1.492 (4)	C7—C8	1.537 (5)
N2—H2AN	0.9000	C8—H8A	0.9600
N2—H2BN	0.9000	C8—H8B	0.9600
N3—C6	1.481 (4)	C8—H8C	0.9600
N3—H3AN	0.9000	C9—H9A	0.9700
N3—H3BN	0.9000	C9—H9B	0.9700
N4—C9	1.488 (4)	C10—H10A	0.9600
N4—H4AN	0.9000	C10—H10B	0.9600
N4—H4BN	0.9000	C10—H10C	0.9600
C1—C2	1.530 (5)		
			
N3—Cr—N2	92.17 (10)	N1—C1—C2	114.6 (3)
N3—Cr—N4	88.82 (10)	N1—C1—H1A	108.6
N2—Cr—N4	179.01 (11)	C2—C1—H1A	108.6
N3—Cr—N1	179.46 (11)	N1—C1—H1B	108.6
N2—Cr—N1	87.78 (11)	C2—C1—H1B	108.6
N4—Cr—N1	91.24 (11)	H1A—C1—H1B	107.6
N3—Cr—Cl2	89.04 (8)	C4—C2—C5	111.9 (3)
N2—Cr—Cl2	92.25 (9)	C4—C2—C1	111.2 (3)
N4—Cr—Cl2	87.65 (9)	C5—C2—C1	111.7 (3)
N1—Cr—Cl2	90.42 (8)	C4—C2—C3	108.8 (3)
N3—Cr—Cl1	89.95 (8)	C5—C2—C3	106.4 (3)
N2—Cr—Cl1	88.28 (9)	C1—C2—C3	106.6 (3)
N4—Cr—Cl1	91.84 (9)	C2—C3—H3A	109.5
N1—Cr—Cl1	90.59 (8)	C2—C3—H3B	109.5
Cl2—Cr—Cl1	178.87 (4)	H3A—C3—H3B	109.5
O3—Cl3A—O2	119.8 (4)	C2—C3—H3C	109.5
Cl3B—Cl3A—O4A	170.0 (5)	H3A—C3—H3C	109.5
O3—Cl3A—O4A	98.6 (4)	H3B—C3—H3C	109.5
O2—Cl3A—O4A	103.2 (4)	C2—C4—H4A	109.5
O3—Cl3A—O1	112.9 (3)	C2—C4—H4B	109.5
O2—Cl3A—O1	109.9 (3)	H4A—C4—H4B	109.5
O4A—Cl3A—O1	111.3 (3)	C2—C4—H4C	109.5
O3—Cl3A—O4B	70.5 (4)	H4A—C4—H4C	109.5
O2—Cl3A—O4B	77.1 (4)	H4B—C4—H4C	109.5
O4A—Cl3A—O4B	166.7 (4)	N2—C5—C2	114.0 (3)
O1—Cl3A—O4B	80.6 (3)	N2—C5—H5A	108.8
O3—Cl3B—O1	121.3 (4)	C2—C5—H5A	108.8
O3—Cl3B—O4B	96.3 (5)	N2—C5—H5B	108.8
O1—Cl3B—O4B	110.6 (4)	C2—C5—H5B	108.8
O3—Cl3B—O2	117.1 (4)	H5A—C5—H5B	107.7
O1—Cl3B—O2	109.2 (3)	N3—C6—C7	115.0 (3)
O4B—Cl3B—O2	98.8 (6)	N3—C6—H6A	108.5
O3—Cl3B—O4A	74.2 (3)	C7—C6—H6A	108.5
O1—Cl3B—O4A	85.3 (3)	N3—C6—H6B	108.5
O4B—Cl3B—O4A	164.1 (5)	C7—C6—H6B	108.5
O2—Cl3B—O4A	75.2 (3)	H6A—C6—H6B	107.5
C1—N1—Cr	119.62 (19)	C10—C7—C6	111.3 (3)
C1—N1—H1AN	107.4	C10—C7—C9	111.2 (3)
Cr—N1—H1AN	107.4	C6—C7—C9	112.3 (3)
C1—N1—H1BN	107.4	C10—C7—C8	108.8 (3)
Cr—N1—H1BN	107.4	C6—C7—C8	106.7 (3)
H1AN—N1—H1BN	106.9	C9—C7—C8	106.2 (3)
C5—N2—Cr	119.9 (2)	C7—C8—H8A	109.5
C5—N2—H2AN	107.3	C7—C8—H8B	109.5
Cr—N2—H2AN	107.3	H8A—C8—H8B	109.5
C5—N2—H2BN	107.3	C7—C8—H8C	109.5
Cr—N2—H2BN	107.3	H8A—C8—H8C	109.5
H2AN—N2—H2BN	106.9	H8B—C8—H8C	109.5
C6—N3—Cr	119.9 (2)	N4—C9—C7	113.7 (3)
C6—N3—H3AN	107.3	N4—C9—H9A	108.8
Cr—N3—H3AN	107.3	C7—C9—H9A	108.8
C6—N3—H3BN	107.3	N4—C9—H9B	108.8
Cr—N3—H3BN	107.3	C7—C9—H9B	108.8
H3AN—N3—H3BN	106.9	H9A—C9—H9B	107.7
C9—N4—Cr	119.9 (2)	C7—C10—H10A	109.5
C9—N4—H4AN	107.4	C7—C10—H10B	109.5
Cr—N4—H4AN	107.4	H10A—C10—H10B	109.5
C9—N4—H4BN	107.4	C7—C10—H10C	109.5
Cr—N4—H4BN	107.4	H10A—C10—H10C	109.5
H4AN—N4—H4BN	106.9	H10B—C10—H10C	109.5
			
O2—Cl3A—Cl3B—O3	−123.6 (4)	O1—Cl3A—O2—Cl3B	67.3 (4)
O4A—Cl3A—Cl3B—O3	20 (3)	O4B—Cl3A—O2—Cl3B	−7.5 (5)
O1—Cl3A—Cl3B—O3	123.2 (4)	O3—Cl3B—O2—Cl3A	67.5 (5)
O4B—Cl3A—Cl3B—O3	−80 (3)	O1—Cl3B—O2—Cl3A	−75.3 (4)
O3—Cl3A—Cl3B—O1	−123.2 (4)	O4B—Cl3B—O2—Cl3A	169.2 (6)
O2—Cl3A—Cl3B—O1	113.2 (3)	O4A—Cl3B—O2—Cl3A	4.3 (4)
O4A—Cl3A—Cl3B—O1	−103 (3)	O1—Cl3B—O3—Cl3A	76.7 (5)
O4B—Cl3A—Cl3B—O1	157 (3)	O4B—Cl3B—O3—Cl3A	−164.5 (7)
O3—Cl3A—Cl3B—O4B	80 (3)	O2—Cl3B—O3—Cl3A	−61.3 (5)
O2—Cl3A—Cl3B—O4B	−43 (3)	O4A—Cl3B—O3—Cl3A	2.4 (4)
O4A—Cl3A—Cl3B—O4B	100 (4)	O2—Cl3A—O3—Cl3B	72.8 (5)
O1—Cl3A—Cl3B—O4B	−157 (3)	O4A—Cl3A—O3—Cl3B	−176.6 (6)
O3—Cl3A—Cl3B—O2	123.6 (4)	O1—Cl3A—O3—Cl3B	−59.0 (5)
O4A—Cl3A—Cl3B—O2	143 (3)	O4B—Cl3A—O3—Cl3B	11.2 (5)
O1—Cl3A—Cl3B—O2	−113.2 (3)	N2—Cr—N1—C1	−41.0 (2)
O4B—Cl3A—Cl3B—O2	43 (3)	N4—Cr—N1—C1	138.9 (2)
O3—Cl3A—Cl3B—O4A	−20 (3)	Cl2—Cr—N1—C1	51.3 (2)
O2—Cl3A—Cl3B—O4A	−143 (3)	Cl1—Cr—N1—C1	−129.2 (2)
O1—Cl3A—Cl3B—O4A	103 (3)	N3—Cr—N2—C5	−137.9 (2)
O4B—Cl3A—Cl3B—O4A	−100 (4)	N1—Cr—N2—C5	41.6 (2)
O3—Cl3B—O4B—Cl3A	78 (3)	Cl2—Cr—N2—C5	−48.8 (2)
O1—Cl3B—O4B—Cl3A	−155 (3)	Cl1—Cr—N2—C5	132.2 (2)
O2—Cl3B—O4B—Cl3A	−41 (2)	N2—Cr—N3—C6	−141.5 (2)
O4A—Cl3B—O4B—Cl3A	25.7 (16)	N4—Cr—N3—C6	38.6 (2)
O3—Cl3A—O4B—Cl3B	−96 (3)	Cl2—Cr—N3—C6	126.3 (2)
O2—Cl3A—O4B—Cl3B	135 (3)	Cl1—Cr—N3—C6	−53.2 (2)
O4A—Cl3A—O4B—Cl3B	−132 (3)	N3—Cr—N4—C9	−39.7 (2)
O1—Cl3A—O4B—Cl3B	22 (2)	N1—Cr—N4—C9	140.8 (2)
O3—Cl3A—O4A—Cl3B	19 (3)	Cl2—Cr—N4—C9	−128.8 (2)
O2—Cl3A—O4A—Cl3B	142 (3)	Cl1—Cr—N4—C9	50.2 (2)
O1—Cl3A—O4A—Cl3B	−100 (3)	Cr—N1—C1—C2	59.1 (3)
O4B—Cl3A—O4A—Cl3B	53 (3)	N1—C1—C2—C4	60.6 (4)
O3—Cl3B—O4A—Cl3A	−160 (3)	N1—C1—C2—C5	−65.2 (4)
O1—Cl3B—O4A—Cl3A	76 (3)	N1—C1—C2—C3	179.0 (3)
O4B—Cl3B—O4A—Cl3A	−105 (4)	Cr—N2—C5—C2	−60.0 (4)
O2—Cl3B—O4A—Cl3A	−35 (3)	C4—C2—C5—N2	−60.1 (4)
O3—Cl3B—O1—Cl3A	−75.2 (5)	C1—C2—C5—N2	65.2 (4)
O4B—Cl3B—O1—Cl3A	173.4 (8)	C3—C2—C5—N2	−178.8 (3)
O2—Cl3B—O1—Cl3A	65.8 (4)	Cr—N3—C6—C7	−56.9 (3)
O4A—Cl3B—O1—Cl3A	−6.8 (4)	N3—C6—C7—C10	−60.2 (4)
O3—Cl3A—O1—Cl3B	59.8 (5)	N3—C6—C7—C9	65.2 (4)
O2—Cl3A—O1—Cl3B	−76.8 (5)	N3—C6—C7—C8	−178.8 (3)
O4A—Cl3A—O1—Cl3B	169.6 (6)	Cr—N4—C9—C7	58.7 (3)
O4B—Cl3A—O1—Cl3B	−4.3 (5)	C10—C7—C9—N4	59.8 (4)
O3—Cl3A—O2—Cl3B	−65.8 (5)	C6—C7—C9—N4	−65.7 (4)
O4A—Cl3A—O2—Cl3B	−173.9 (6)	C8—C7—C9—N4	178.0 (3)

### Hydrogen-bond geometry (Å, °)

**Table:**

*D*—H···*A*	*D*—H	H···*A*	*D*···*A*	*D*—H···*A*
N1—H1BN···O2^i^	0.90	2.29	3.030 (5)	139
N2—H2AN···O3^ii^	0.90	2.23	3.099 (6)	162
N2—H2AN···O4A^ii^	0.90	2.42	3.183 (6)	143
N2—H2BN···O4B	0.90	2.36	3.217 (9)	159
N3—H3AN···O4A^ii^	0.90	2.60	3.482 (7)	168
N4—H4BN···O2^i^	0.90	2.14	3.030 (5)	172
N4—H4AN···O4A^iii^	0.90	2.54	3.403 (8)	161
N1—H1AN···Cl2^iv^	0.90	2.68	3.525 (3)	156
N3—H3BN···Cl1^ii^	0.90	2.69	3.533 (3)	156

Symmetry codes: (i) *x*, −*y*+1/2, *z*+1/2; (ii) *x*−1, *y*, *z*; (iii) *x*−1, −*y*+1/2, *z*+1/2; (iv) *x*+1, *y*, *z*.
